# Berberis Aquifolium

**Published:** 1878-10-20

**Authors:** 


BERBERIS AQUIFOLIUM.

A sample of the above remedy, kindly sent us by Messrs Park, Davis & Co. *Manufacturing chemist* at Detroit, has been tested, in an old and obstinate case of scrofula in a little girl, with very satisfactory results. The properties of this drug we mentioned in advertisement on first inside cover of our Journal.

